# Family members´ experiences of the end-of-life care environments in acute care settings – a photo-elicitation study

**DOI:** 10.1080/17482631.2018.1511767

**Published:** 2018-09-03

**Authors:** Yvonne Hajradinovic, Carol Tishelman, Olav Lindqvist, Ida Goliath

**Affiliations:** a Palliative Education & Research Centre, Region Östergötland, Vrinnevi hospital, Norrköping, Sweden; b Sophiahemmet University, Department of Nursing Science, Stockholm, Sweden; c Division of Innovative Care Research, Department of Learning, Informatics, Management and Ethics, Karolinska Institutet, Stockholm, Sweden; d The Center for Rural Medicine, Storuman, Västerbottens county council (VLL); e Stockholm Health Care Services (SLSO), Stockholms country council (SLL), Stockholm, Sweden; f Department of Nursing, Umeå University, Umeå, Sweden; g Ersta hospital, Hospice, Stockholm, Sweden

**Keywords:** Acute care, care environment, end-of-life, family members, hospital, photo-elicitation, visual research methods

## Abstract

Purpose: This article explores experiences of the acute-care environment as a setting for end-of-life (EoL) care from the perspective of family members of a dying person. Method: We used participant-produced photographs in conjunction with follow-up interviews with nine family members to persons at the EoL, cared for in two acute-care settings. Results: The interpretive description analysis process resulted in three constructed themes—Aesthetic and un-aesthetic impressions, Space for privacy and social relationships, and Need for guidance in crucial times. Aspects of importance in the physical setting related to aesthetics, particularly in regard to sensory experience, and to a need for enough privacy to facilitate the maintenance of social relationships. Interactions between the world of family members and that of professionals were described as intrinsically related to guidance about both the material and immaterial environment at crucial times. Conclusion: The care environment, already recognized to have an impact in relation to patients, is concluded to also affect the participating family members in this study in a variety of ways.

## Introduction

In Sweden, about 42% of all deaths in 2012 took place in acute-care hospitals (Hakanson, Ohlen, Morin, & Cohen, 2015). In Broad et al.’s study (Broad et al., 2013), half of the 16 million deaths reported from 36 countries occurred in hospitals. Acute-care hospitals are thus a common place of death internationally; however they are generally fast-paced, high-tech environments, with care of the dying neither a primary focus nor prioritized (Bloomer, Endacott, O’Connor, & Cross, 2013; Chan, Macdonald, Carnevale, & Cohen, 2017). Several researchers point to deficits in quality end-of-life (EoL) care in acute-care hospitals (Al-Qurainy, Collis, & Feuer, 2009; Oliver & O’Connor, 2015; Reyniers, Houttekier, Cohen, Pasman, & Deliens, 2014), with both organizational and environmental factors contributing to a lack of support for dying persons and their family members (Sheward, Clark, Marshall, & Allan, 2011; Virdun, Luckett, Lorenz, Davidson, & Phillips, 2017).

Aside from intensive care environments (e.g., Bloomer, Endacott, Copnell, & O’Connor, 2016; Fridh, Forsberg, & Bergbom, 2009; Salins, Deodhar, & Muckaden, 2016; Wetzig & Mitchell, 2017), we have found little research on family members’ needs in or experiences of acute-care hospitals as settings for EoL care. Research on family members in acute-care settings mainly concerns problems experienced by family members, e.g., experience of patients’ symptom burden and management, communication and relationships with professional caregivers, involvement in decision making, or their own needs for care (Robinson, Gott, & Ingleton, 2014; Steinhauser, Voils, Bosworth, & Tulsky, 2015; Virdun, Luckett, Davidson, & Phillips, 2015). The tendency to focus on life-sustaining treatment may encourage a disengagement from, and diminish recognition of, the acute-care environment as a setting for EoL care (Bloomer et al., 2013; Chan et al., 2017).

It is well established that family members are physically and psychologically burdened by the dying and death of someone close, with increased risks for their own health (Masterson et al., 2015; Sampson et al., 2016). Hospitals as a place of death may exacerbate such problems, as they are associated with lower quality of life for patients, but also higher risks for prolonged grief in bereaved family caregivers (Wright et al., 2010).

In a previous study from this research group (anonymized reference), our analysis of patient-generated photographs with subsequent interviews underscores the variety of ways in which surroundings play a central role in physical, functional and social wellbeing from the perspective of people living in what was likely to be their last residence, either hospice, home care or a residential care home.

There is a broad literature on evidence-based hospital design and healing spaces in a variety of health care settings (e.g., DuBose, MacAllister, Hadi, & Sakallaris, 2018; Fröst & Hammarling, 2017; Maben et al., 2015). Numerous efforts have been made to study patient and staff outcomes in relation to specific design characteristics or interventions, including room occupancy (e.g., Maben et al., 2016), the acoustic environment (e.g., Shield, Shiers, & Glanville, 2016), visual contact with nature/landscapes (e.g., Ulrich, 1979), sound and lighting (e.g., Voigt et al., 2017), ergonomic design (e.g., Knibbe & Waaijer, 2012), and the work environment (e.g., Lee & Scott, 2018). Robust research indicates that the design of the care environment has implications for patient safety, communication, coping and experience of stress (Andrade, Lima, Devlin, & Hernandez, 2016; Ulrich et al., 2008). Hence, the surroundings may either facilitate or obstruct quality EoL care (Beckstrand, Rasmussen, Luthy, & Heaston, 2012; Sagha Zadeh et al., 2018; Slatyer, Pienaar, Williams, Proctor, & Hewitt, 2015). Supportive features in the institutional hospital environment have been identified as options for social interaction and privacy (Brereton et al., 2012), but the lack of these options, and the environment being too busy and/or noisy have been pointed out by both patients, relatives, staff and as environmental deficiencies (Pincombe, Brown, & McCutcheon, 2003; Rigby, Payne, & Froggatt, 2010; Robinson et al., 2014). Stajduhar and Davies (2005) found that family members’ negative experiences of institutional care and particularly acute care which they described as depersonalized and paternalistic, was main reasons for caregivers deciding to care for a dying person at home (see also Wennman-Larsen & Tishelman, 2002).

Hospitals continue to be major settings for EoL care and further knowledge about experiences of these settings is thus needed (Reyniers et al., 2014), to improve evidence and practice (Grande, 2009). However, as noted, studies focusing on family members’ experiences of the acute-care environment during ongoing EoL care seem to be rare. The aim of this study is therefore to explore experiences of the acute-care environment as a setting for EoL care from the perspective of family members of a dying person. This study derives from the DöBra research programme’s (in Swedish this term is a pun, meaning both “dying well” and “awesome” (anonymized reference), line of action research “Space and place in EoL care”, working towards more supportive EoL care environments.

## Method

### Study design and study context

In this qualitative, inductive study, we explored family members’ experiences by using participant-produced photographs in conjunction with follow-up interviews analyzed with interpretive description (Thorne, 2016). The data collection approach, also referred to as “photo-elicitation” or “photovoice”, terms often used interchangeably, was first described in 1997 as a form of community-based participatory research used to address health and social justice issues by engaging participants in the research process as active documentarians, commentators, and agents of social and political change (Catalani & Minkler, 2010). It has been found useful in exploring issues that may be difficult to verbalize (Balmer, Griffiths, & Dunn, 2015), especially among vulnerable populations (Bugos et al., 2014), and when studying palliative and EoL care (anonymized reference, Moore, Carter, Hunt, & Sheikh, 2013)). This project has been approved by the Regional Ethical Review Board in Stockholm (Dnr Ö 29–2012).

The study took place at two acute-care units providing care for patients with lung diseases. One unit had 15 patient beds, and was situated in a large university hospital in a major city. The other 21-bed unit was in a local hospital in a smaller city; both were in southern-mid Sweden. In 2014, 42 respectively 105 deaths occurred in these units. In spring 2015, both units had an average in-patient stay of seven and a half days.

### Recruitment and data collection

Purposive sampling was used (Thorne, 2016) to recruit participants, who met the inclusion criteria: family members aged >18 years of patients in a palliative phase of a life-threatening disease, on-going inpatient care, no known cognitive impairments, and able to read and speak Swedish. We aimed to recruit participants’ with experiential knowledge from prolonged time spent with their severely ill family members in the acute care environment, who were willing to use their time and engage in the research; take photographs of the care environment and thereafter share their reflections, thoughts and experiences. Studies inspired by interpretive description tend to have a sample size of five to 30 participants (Thorne, 2016). In-depth information from a small number of information-rich people has been found to generate a large amount of useable and relevant data (Patton, 2015).

Prior to data collection, the study was presented at a staff meeting at each unit. One or more nurses agreed to inform eligible participants about the study. These contact nurses were also asked to first clinically assess patients’ phase of illness, deterioration and/or physical signs of progressing illness, to help identify people in a late palliative phase with limited life span and/or impending death. No specific instruments were used in the assessment, but the first author, a palliative care consultant nurse, supported the contact nurses in their clinical judgements as staff at the study settings had some difficulty in identifying persons in the EoL.

After receiving information about potential participants from the contact nurses, the first author telephoned the family member to provide verbal information about the study, and ask about interest in participation. The information highlighted the voluntary nature of participation, the possibility to withdraw from the study without explanation, and that withdrawal would not affect the participant or patient care in any way. After agreeing to participate, the family member was lent a digital camera and an interview was booked for the following day. The family members were asked to take three photographs each of that which they found important or meaningful to them in the acute-care environment. The number of photographs was chosen to enable participants to depict different aspects of the environment that were salient to them around the time for their participation in the study, without overburdening them (Anonymized reference, Balmer et al., 2015). The participants were also informed about the need for obtaining informed consent if they took photographs including other people in the unit (Catalani & Minkler, 2010).

Data collection was performed between November 2014 and April 2015. Individual interviews took place in a quiet room in or close to the unit and were performed by the first author. In total, 27 photographs, three per person, were produced by the nine participants. At the onset of each interview, the photographs were transferred to a computer screen. Two questions were used to stimulate conversation, “What is this picture of?” and “Why is it meaningful to you?”, followed by further follow-up questions dependent on what was told. Family members were also asked if they would like to give a title to each photograph (Radley & Taylor, 2003). The interviews were digitally audio-recorded and generally lasted 30–90 minutes, with one exception. This interview was recorded for 9 minutes but as the family member was uncomfortable with audio-recording but still wished to participate, the remaining approximately 10 minutes was documented by notes only. The interviews were transcribed verbatim by the first author.

Nine participants took part in the study. Six were women and three were men, of whom four were daughters, one was a son, and four were spouses; their ages ranged from 23 to 63 years. All men and three women had university degrees and the remaining three women were high graduates. One participant lived in a rural area, and the rest in urban areas. The time family members spent in the acute-care unit with the patient varied from 2 to 24 hours/day. The sick family members were in late stages of lung or kidney diseases, and had been in contact with their present hospital unit for between 1 week prior to interview, to having had intermittent admissions over a period of 1.5 years.

### Analysis

Interpretive description guided design of the concurrent data collection and analysis (Thorne, 2016). Each interview with accompanying photographs was seen as an analytic unit, that is the photographs alone were not subject to analysis of their content, but were only viewed conjunction with the interview data as follows (Anonymized reference). The first author initially listened to the audio-recorded interviews, and simultaneously read transcripts to correct errors in transcription and obtain a sense of the interview as a whole. Thereafter each interview was carefully read through while viewing the participant-produced photographs and memos of preliminary ideas and themes were written. Inductive broad-based coding was then performed, and text with similar content, e.g., related to sensory impressions, grouped together (Thorne, 2016). During analysis, initial codes were examined and discussed among the authors and, in the process of identifying variations and relationships in the text, were further developed. The first and the last author worked together throughout the analysis, repeatedly discussing the analytical process and findings with the second and third author to validate and reach agreement on interpretation and themes. The analysis process resulted in three constructed themes—Aesthetic and un-aesthetic impressions; Space for privacy and social relationships, and Need for guidance in crucial times—describing different aspects of the experiences of family members in the acute-care hospital environments. The results are presented in relation to each theme.

## Results

### Aesthetic and un-aesthetic impressions

**Figure 1.**
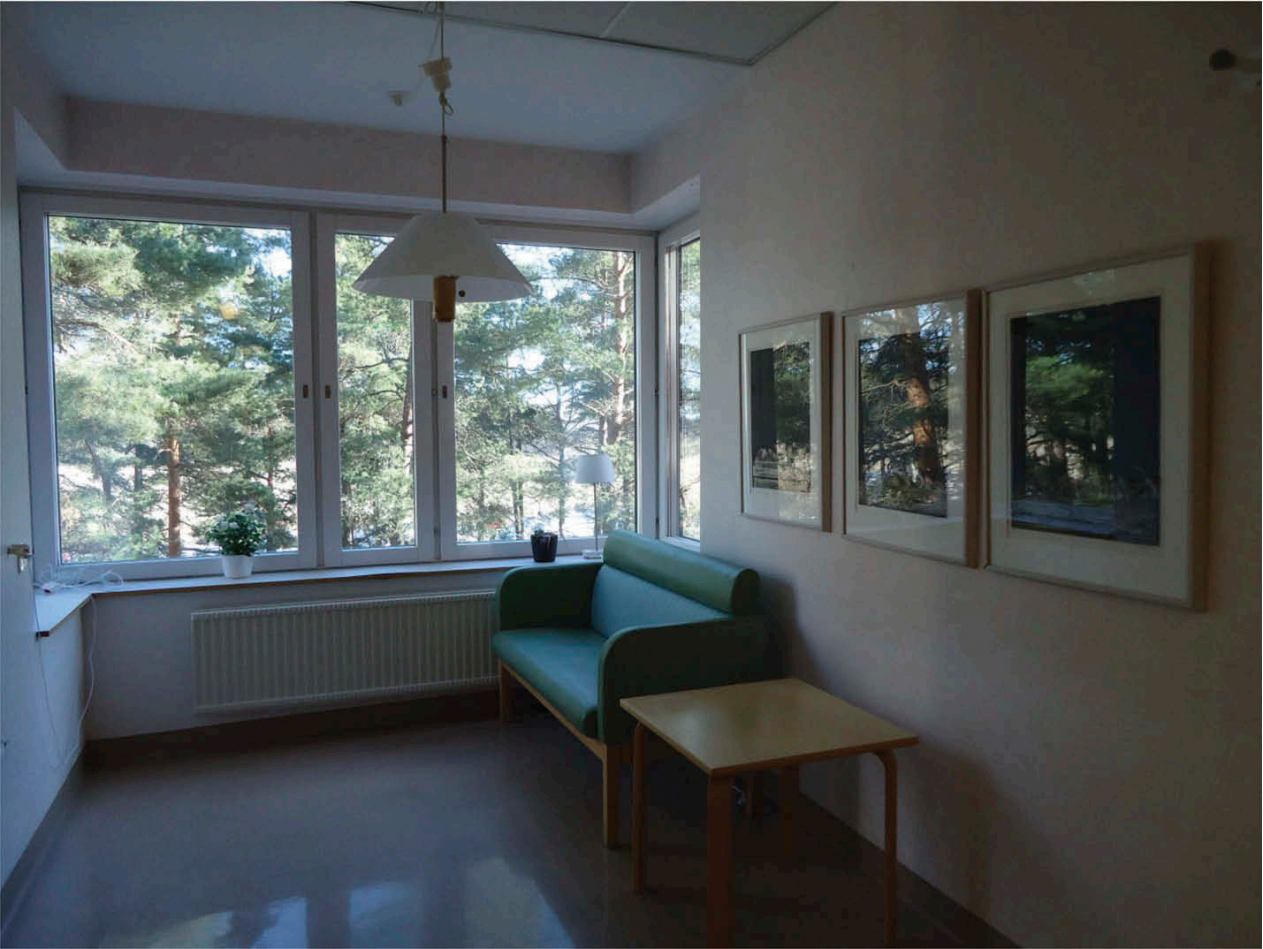
Corner of a hospital corridor

This theme includes descriptions of different aesthetic impressions that contributed to experiences of the acute-care environment. These were exemplified by participants’ photographs of equipment, furnishings, decorations and art, including colour and fabrics. The aesthetic impressions described were clearly related to sensory experiences, primarily sight and sound. One family member talked about a picture she took, shown in Figure 1, depicting a corner of a corridor. She said this corner felt carefully arranged, basing this on the colours of the furniture, the art and the view. She also underscored that being able to see nature and life outside the window gave her a moment of respite.

**Figure 2.**
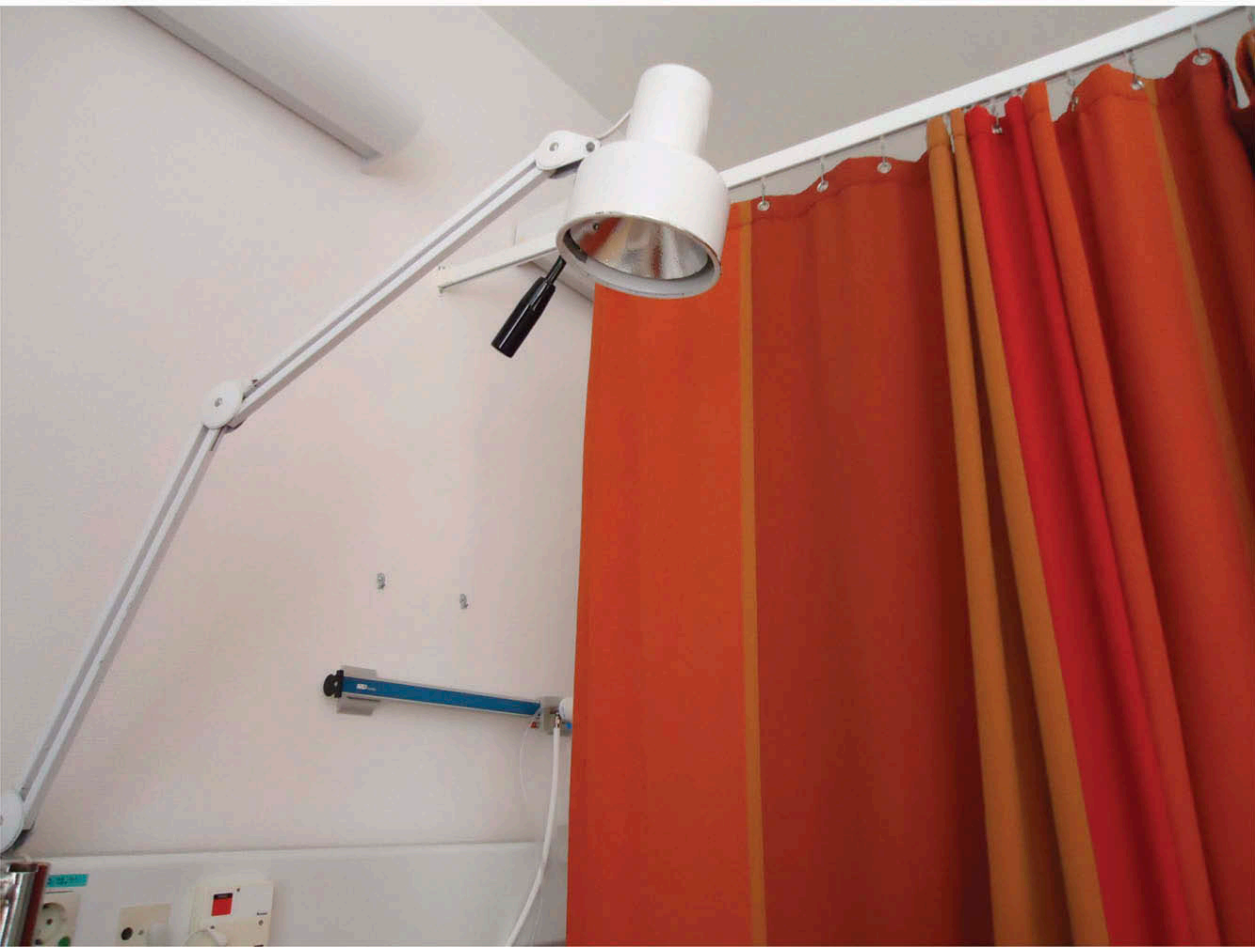
Red curtain between two beds in a patient room

Several family members emphasized that the colours in patient rooms, waiting rooms, day rooms and corridors, influenced their overall experience of the care environment. Colours experienced as warm and joyful were said to relieve distress, while colours described as glaring and cold were experienced as enhancing anxiety, feelings of physical chilliness and even confusion. However, there was little consensus about which particular colours was defined as warm or cold. Several family members talked about their feelings about colours in relation to textiles, such as curtains, bed linen or a personal blanket. Looking at her photograph of a thick, red curtain between the two beds in the patient room, shown in Figure 2, a spouse explained that when she saw this curtain she experienced breathing difficulties and a sense of discomfort from not being able to see if anyone was behind it. Another participant, a patient’s son, pointed out that beyond colour and art, lighting also contributed to an aesthetic environment:

”…it is very important, what is the lighting like? What are the colours like? What are the pictures like? The aesthetics of the environment around you, that can make you feel, feel at ease. It´s common knowledge that intense lighting, bare walls, white, …the classic hospital environment… can be pretty scary.”

This family member emphasized several specific aesthetic features that contributed to his feelings of being either at ease or frightened. Several participants spoke of the need for improvements in lighting, with reference to the cold light from fluorescent ceiling lights. One husband took a photograph of a dim, poorly lit room, using descriptors such as “terribly bad” and “dreary” about the light. He stated his clear preference for warm and functional lighting, e.g., for reading, at the same time saying that such lighting could not be found in the room. Other family members described lacking the possibility to adjust lighting according to different needs, for example dimming the lights.

**Figure 3.**
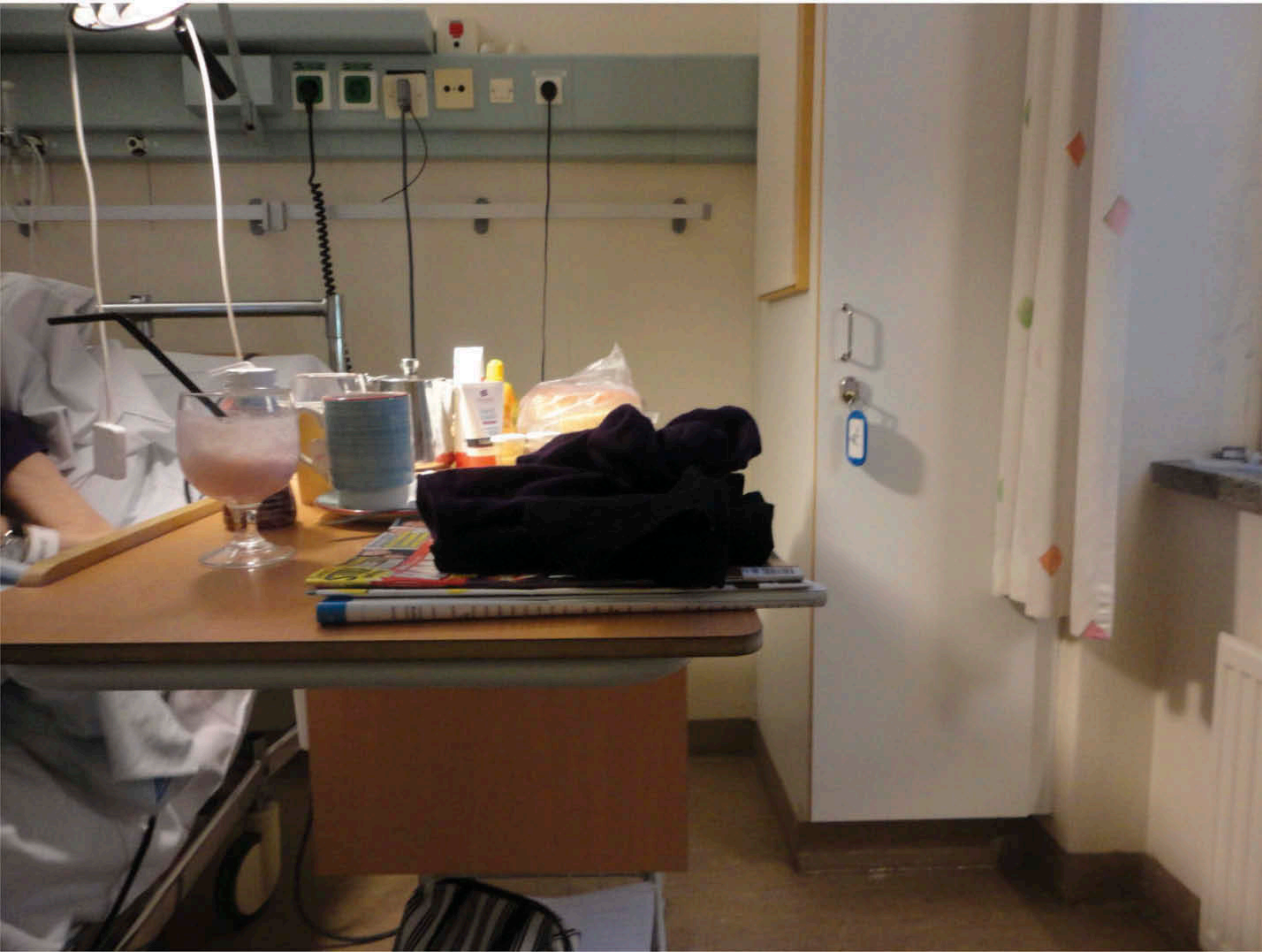
Bedside table

Several participants photographed and talked about art and other things they found beautiful on the unit; as one participant said: “It helps to clear your head and then you can think more logically”. In contrast, another family member talked about a piece of art depicting the devil, as an example of art she did not find positive or elevating. Order and cleanliness were also aesthetic factors that were described as indicating consideration of and attention to the care environment; however this was a topic of conversation only when perceived to be absent. Participants gave several examples in which such consideration was lacking, e.g., dirty floors and toilets, stains on tables, and an assortment of worn or broken things on the unit. Some family members said that staff did not care or pay attention either to details or to the overall orderliness of the unit, which made them question the quality of care. One daughter photographed her mother’s bedside table, shown in Figure 3 and described as overfull; she shared her idea of increasing functionality through a more flexible table size that could help create order.

Other aesthetic impressions related to stories about the sound environment, often inspired by photographs depicting technical devices. Repeated beeping and time delays in staff responding to alarms were said to be highly stressful for the family members. A wife photographed intravenous equipment and named the picture “The drip from hell”:

“What can I say? Disgust, discomfort and a little impatience too. Damn! Can´t they come and turn it off?! It eats away at you somehow, that sound. It’s so monotonous and then you know, it’s quiet a while and then it starts again like, like … sounds, unpleasant, nasty noises, you want to, yes, to escape. You want to get away from it…”

**Figure 4.**
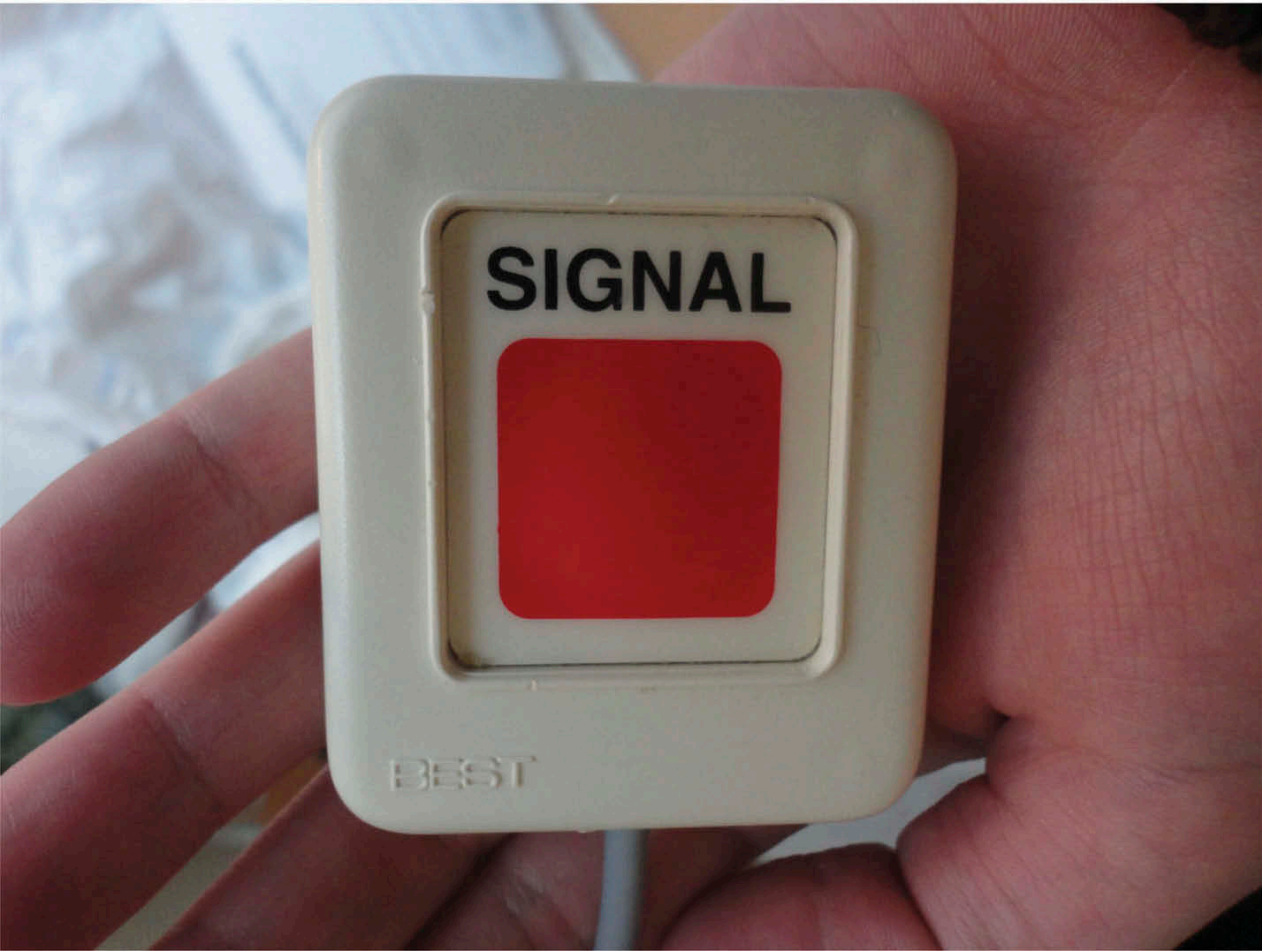
Alarm button

This wife found the equipment and the noise that she described as dominating day and night particularly upsetting since it reminded her of medical technical errors experienced earlier in her husband´s sickness trajectory. Another technical device mentioned by several family members was the alarm button. It was said to be problematic that signals intended to convey different meanings and messages could only take one form, as one family member exemplified: …”This button says: ’I would like a napkin, a straw, or I can´t breathe, I´m dying‘”. This daughter named her photograph (see Figure 4); “The stress alarm button” speaking of how distressing it was for her to not know how long it would take before somebody would respond to the alarm.

Other disturbing noises were said to come from other people in shared rooms, with family members describing distress at not being able to get away from these sounds. A family member who had brothers who used hearing aids, described difficulties when communicating, since their hearing aids amplified sound. Explaining his photograph of a television shared by several patients, he spoke about how it affected the sound environment and obstructed conversations.

### Space for privacy and social relationships

**Figure 5.**
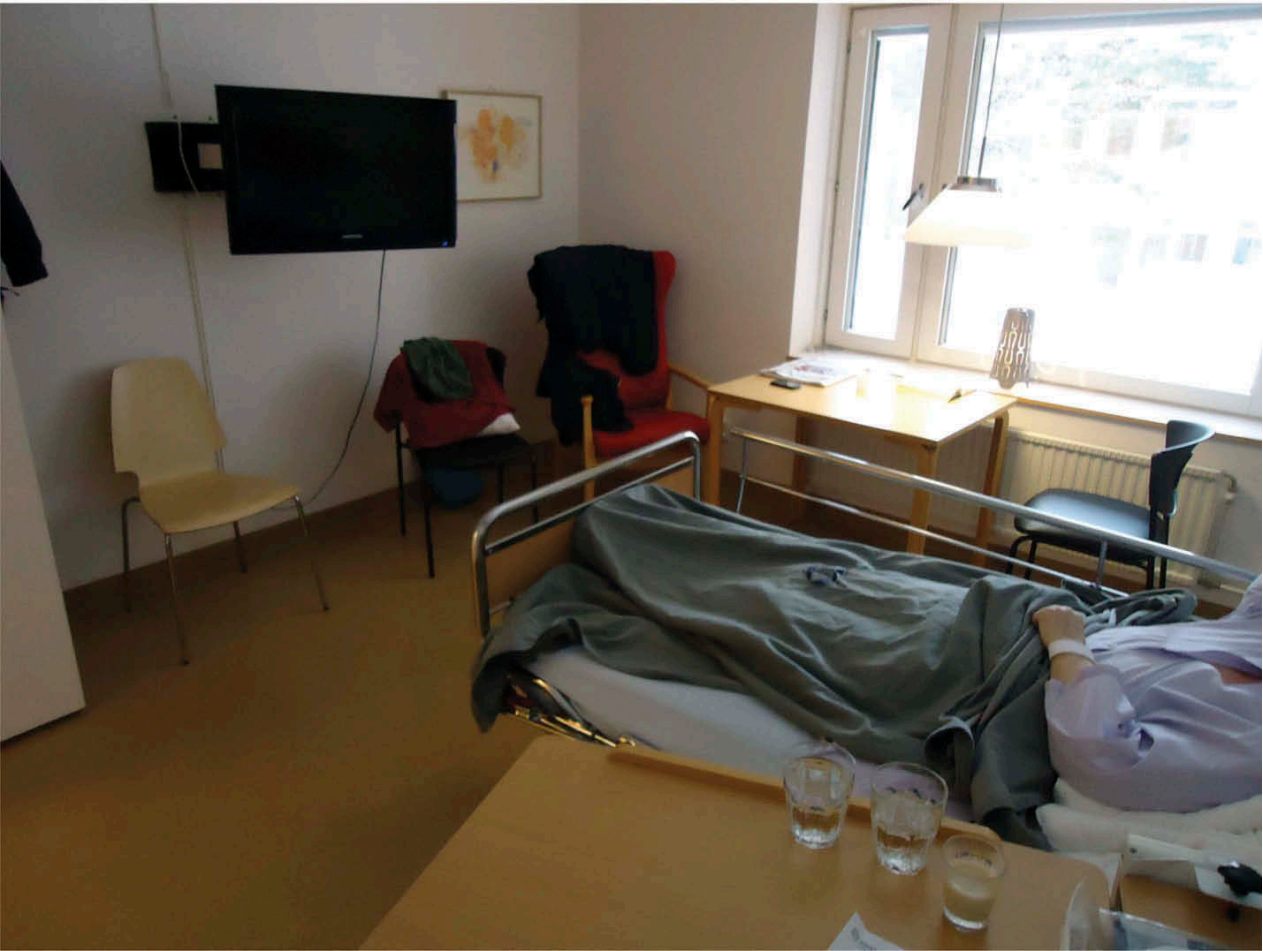
Interior of a single-patient room

In these data, the need for space for privacy and maintaining social relationships was a reoccurring theme, emphasized by most family members. Their stories were illustrated by photographs depicting the interiors of single rooms, as in Figure 5, or of partitions between beds. Electronic devices were also found in many photographs, explained as means for maintaining social relationships and enabling what was said to be, vital contact with everyday life. One husband used his photograph of a mobile phone to emphasize “Internet is as important as air and water”. His lack of internet access in the hospital seemed to increase his sense of being cut-off from everyday life.

A number of participants highlighted their need to communicate about EoL experiences with their other family members. When there was more than one family member, being able to talk to each other about information from staff, the illness, the future, and everyday topics was described as helpful in their situation. Flexible visiting hours and the possibility to stay overnight made it easier to adapt visits to the sick person´s needs. Single rooms were described as meeting a need to use space based on individual preferences, and were said to add a sense of security and control in a situation in which a sense of control was sometimes described as a scarce commodity.

According to one daughter, it was possible to create some kind of everyday life in the otherwise far from ordinary situation in her mother’s single room. A number of family members could visit at the same time, including children, since they did not disturb other patients and, as this daughter said: “It felt like it was more acceptable to laugh and horse around”. Being able to do things together had implications for feeling like a family, as evidenced in this daughter’s mention of how important it would have been for her parents to be able to share meals in her mother’s room:

“I think that it would be very valuable, for example for my father. They’ve [her parents] been married for 55 years. They have shared their meals on most days during these 55 years. He’s here every day, so I think he should be able to buy food and eat it with my mother.”

**Figure 6.**
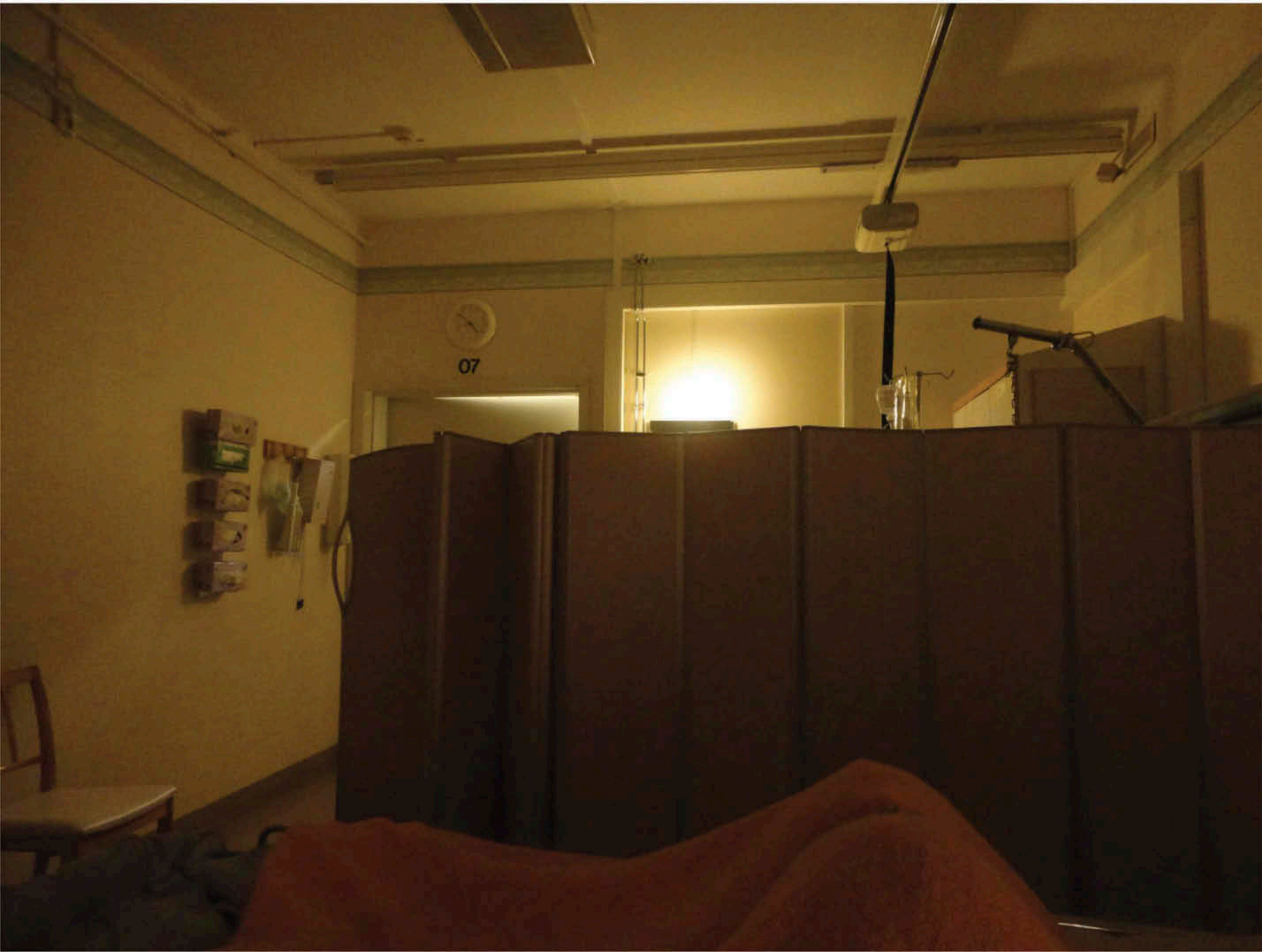
Partition between beds

In this quote, sharing a meal was an example of everyday life routines important for maintaining family relationships. When the dying person had to share a room with others, family members saw this as a hinder, which affected social contact. One son described the double room as a security risk, as he unwillingly heard other patients’ private medical information. This also made him hesitant to talk about private matters with his mother, to avoid exposing them both. He took Figure 6, depicting a moveable partition between his mother´s bed and that of another patient, saying that this did not offer either privacy or tranquillity. Even other participants described their need to talk about the past and the future with their dying family members. One daughter described:

“When you’re in a room where there are four sick people and even if my mother had just recovered to some extent, enough to be transferred to a room with four beds, again, because there was someone who was even more sick who needed her room […] And there was one woman who was so extremely sad and her husband sat there, and he was also really sad. And then, then well, you don´t want to laugh even if they are sitting on the other side of the curtain and all that, well, you want to show them some consideration.”

In contrast to the above situation, another participant talked about her photograph of her mother’s single room as a protective space, sparing her mother the suffering and death of other patients. However, a few family members expressed concern about the potential loneliness of the dying person. The only positive feature described in relation to a shared room was the possibility of the dying person being less lonely, with one adult daughter emphasizing that “sharing should be a choice.” One issue with a shared room, described by family members, was when common conveniences were used by several patients, for example different preferences regarding the television. As one participant said:

“You get into a kind of loyalty conflict…since there is only one remote control, should I take it and make sure my mother can watch what she wants to…or should I be a little more generous and try to convince her to watch sports instead of the Nobel prize dinner?”

### Need for guidance in crucial times

The photographs generated stories about the need for guidance in an institutional acute-care environment during a crucial time of life. The participants’ photographs categorized here depict staff, professional badges, a work station, documentation material, a wall clock, and kitchenware. This theme included stories about guiding signs and signals, and staff’s verbal and non-verbal communication. Family members spoke of lacking guidance to be able to readily understand and orient themselves in the acute-care environment. Written messages such as signs at the entrance door, for example stating visiting hours or other restrictions, were sometimes perceived as reproving. Some participants described a lack of practical information, for example where to find toilets, whereas others expressed a need for advice, e.g., where to go for fresh air, and when it is most suitable to leave the unit with the patient for a break. One daughter called this a “framework for how to act”. The lack of such information seemed to leave family members uncertain about how to behave in this environment.

Small things, such as being greeted on arrival or met with a smile, were described as creating a sense of being seen. Body language and appearance, eye contact, standing still while calmly answering questions, were talked about as indicators of respect. A positive and problem-solving attitude in staff was described by family members as making them feel acknowledged as individuals with needs that are important to meet. One daughter talked about this in relation to her photograph of a nurse and a nursing assistant: “It feels like you’re allowed to ask them questions!” Several family members talked about humour, the need for joy and laughter in encounters with staff. Another daughter took a photograph of two nurses standing close to her mother´s bed, in conversation with her:

“Well, they are so considerate in a really nice way and they really show compassion and concern and they are comforting, and can have a good laugh and well, they ask how we are. They are really great.”

She described that when staff helped her mother to be calm, she could also be calm. Her comments were reinforced by another participant who emphasized how essential trust in staff was, when talking about her photograph which she named “The pathway to mother”. This daughter referred to a phone call from a nurse about her mother’s rapid deterioration, which enabled her to get to the hospital and be present at her bedside at a critical time. In contrast, other participants related more negative experiences of staffs’ lack of perceptiveness and sensitivity, as exemplified by one daughter who spoke of receiving information from doctors who used what she called a “rehearsed manuscript”. Receiving contradictory information was also said to be confusing. Several family members described how doctors discussed important medical issues without presenting an understandable goal, for example when choosing between treatment options. Decisions were made and then sometimes quickly changed without input from the dying person or their family members.

Vague answers and reliance on medical expressions seemed to leave family members with questions and a sense of insecurity. As this family member says:

“…I´ve felt there was a lack of patient care planning or ’What are you thinking now?’, ’What’s the next step?’ or… ’What are you checking now?’ and ’When do you think something new will happen?’ or ’When, about when, do you think you will get the answers to certain things?’… “

**Figure 7.**
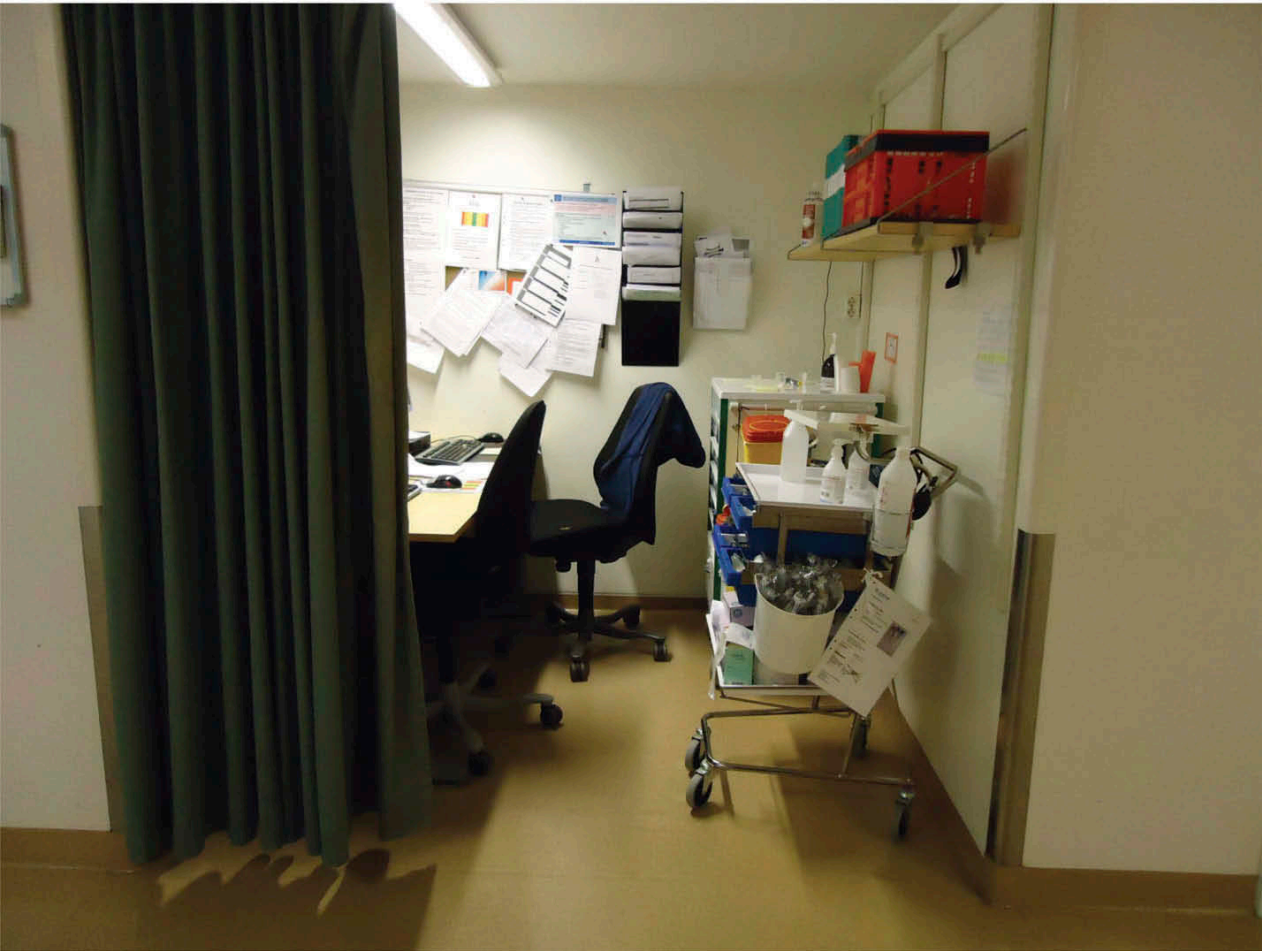
Workstation in the corridor

In spite of all the hospital staff, medical records, equipment and devices, one daughter characterized the situation as “so much information and still so little” in regard to her photograph of a workstation in the corridor, shown in Figure 7. Another daughter also emphasized the importance and use of time, depicting a clock on the wall in one photograph. However, family members also could talk about being engaged in dialogue with staff as part of a team involved in decisions about care.

**Figure 8.**
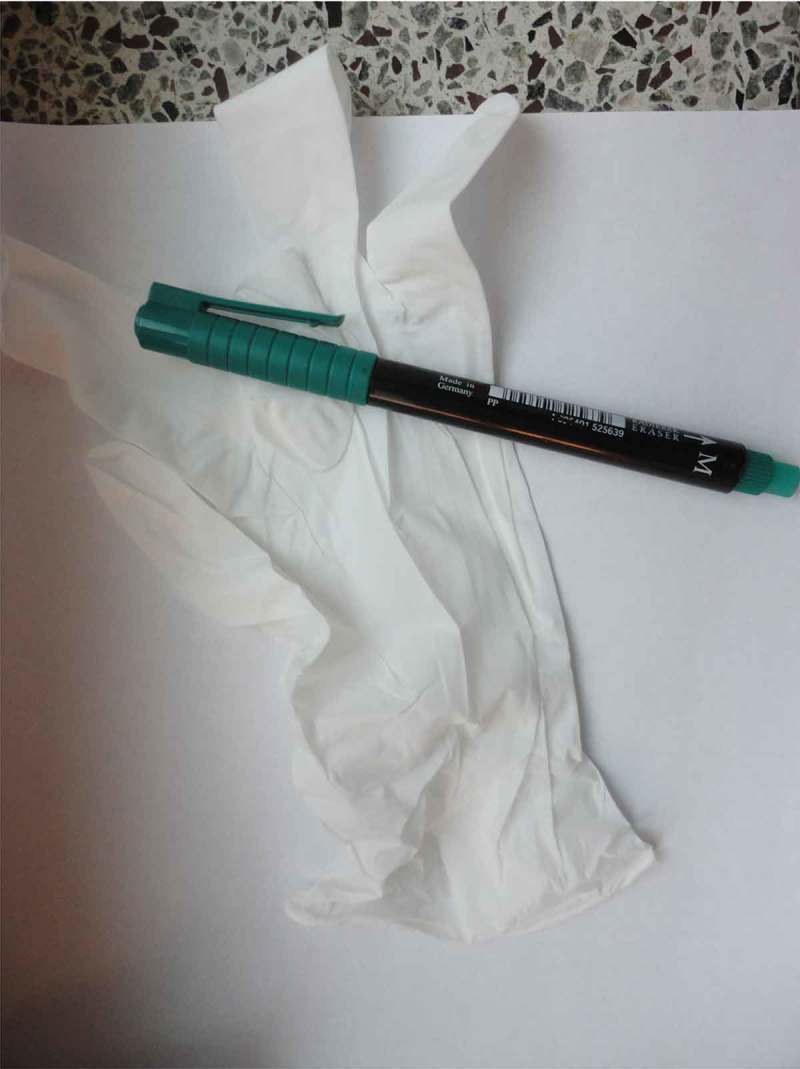
Piece of paper, a rubber glove and a pen

**Figure 9.**
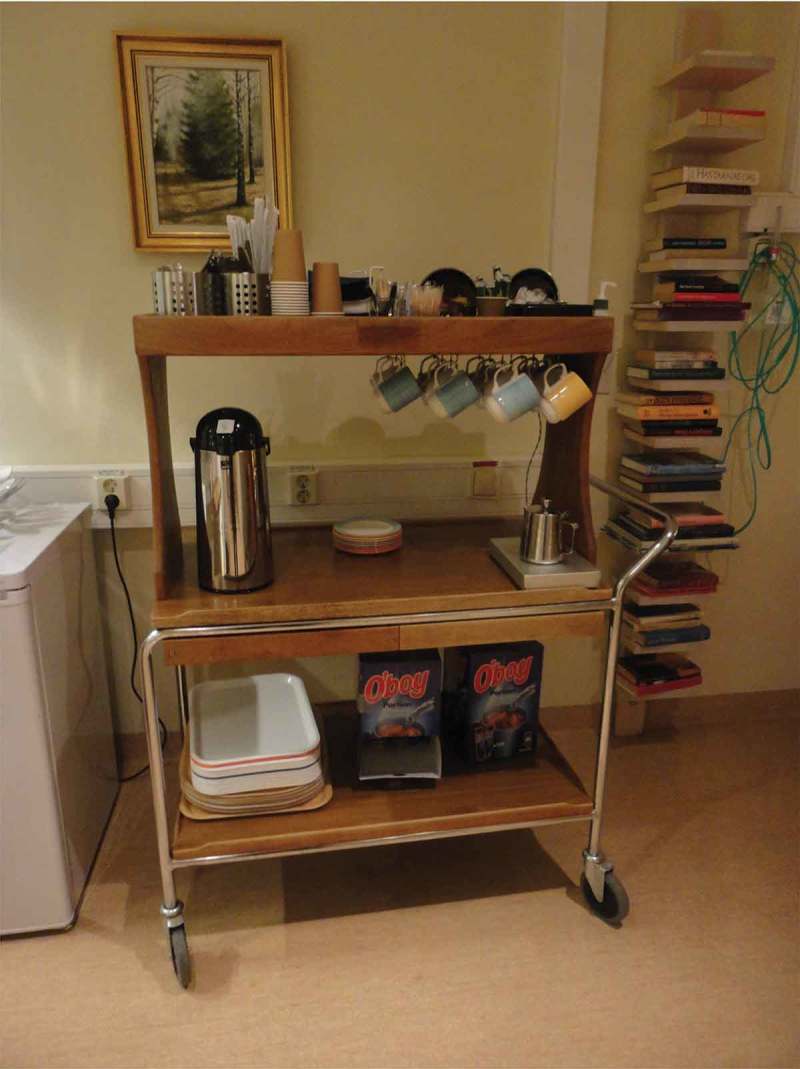
Trolley with beverages in a dayroom

Meeting a lot of different staff members was said to be problematic in a variety of ways. One husband said “It’s like a lottery”, when talking about his photograph of a badge which said “physician” but gave no name. Family members described staff as often stressed and lacking structure in their work, sometimes forgetting important tasks. A daughter summarized this with the title “Unstructured confusion” for her photograph of a piece of paper, a rubber glove and a pen, shown in Figure 8, explaining that staff jotted down important issues on just about anything, including their hands. The lack of communication among staff was said to lead to an experienced need for family members to be physically present, to check and react. Reminding, questioning and objecting to staff actions was mentioned as challenging for the family members. Several participants described feeling like they were obstinate, an inconvenience and/or a disturbance to staff, when they made efforts to take responsibility for the dying family member´s care. On the other hand, staff gestures of hospitality, for example being offered something to eat or drink, were related by several family members as supportive, for example as a husband pointed out in relation to his photograph of a cup of coffee. A son spoke of a trolley with beverages in the dayroom, shown in Figure 9, as a sign of consideration, saying it gave him a chance for a pause outside his mother’s room. This offered moments of respite during his visits to his dying mother.

## Discussion

In this study, family members’ photographs and descriptions of the acute-care environment as a site for ongoing EoL care elicited features that supported or obstructed their wellbeing in these settings. Aspects of importance in the physical setting related to aesthetics, particularly in regard to sensory experience, and to a need for enough privacy to facilitate the maintenance of social relationships. A third area related to interactions between the world of family members and that of professionals, was human interactions between staff and family, which were described as intrinsically related to guidance about both the material and immaterial environment at crucial times.

Entering the setting, a sense of being welcomed and seen as important, was frequently emphasized in the interviews, in terms of experiencing a physical and relational atmosphere of hospitality. In our previous research, we found that the aesthetics of place—sensory experience, atmosphere and beauty—were important in patients’ descriptions of what made them feel supported, cared for, and prioritized in EoL facilities (anonymized reference). Rasmussen and Edvardsson (2007) also found that hospitality was central in how the atmosphere in a unit is experienced, both in acute care and hospice settings. Pallasmaa (2014 p. 21) defines atmosphere as “the overarching perceptual, sensory and emotive impression of a space, setting, or social situation”, and explains that ”it provides the unifying coherence and character of a room, space, place and landscape, or a social encounter”. It should be noted that even in these references, hospitality and atmosphere are not used to refer to the physical environment alone, but also include interactions among people, space and things.

Both voluntary and involuntary interactions with colours, art, textures, materials, spaces and objects added to family members’ multi-sensorial experiences in the care environment. Exploring subjective sensory or sensory-emotional values is part of the study of aesthetics (Zangwill, 2014). Aesthetics is usually described as the study of beauty, relating to arts as something appreciated as pleasing (Paniagua, 2004). Some research (Caspari, Eriksson, & Naden, 2011; Moss & O’Neill, 2014) has shown the importance of aesthetic values for patients’ wellbeing, but these studies did not include family members’ experiences.

Our results indicate that family members were sensitive to both sensory stimulation and the general atmosphere in the acute-care environment, which was perhaps most apparent in discussion of light and sound. Through photographs and discussion of light, the need for individualization in the care environment was addressed as family members in our study spoke about their need to modulate light to different situations. Miwa and Hanyu (2006) emphasize the implications this might have for the care environment, based on their study of how different lighting influences counselling meetings., They found that their research participants spoke more openly in a room with dimmed lighting, seen as relaxed and pleasant, than in one with bright lighting. Sounds seemed to play a different role, as family members in our study spoke only of being negatively affected by sounds, especially repetitive or ongoing alarm noises, designed to gain attention to potential safety risks. This finding is in line with other studies pointing out that sound and noises can increase the risk for additional distress and disturbance (O’Connor et al., 2012; Rigby et al., 2010).

A more subtle type of sensory experience, i.e., a perceived lack of orderliness and cleanliness, was an aspect of an un-aesthetic environment that seemed to enhance negative experiences, also seen in our earlier findings where patients experienced negative aesthetics and a lack of order to be deeply problematic (anonymized reference). Family members’ observations about how the care environments were attended to, suggest that symbols of consideration or lack thereof, were also associated to quality of care and patient safety by them.

Another salient theme in our results is how the EoL setting in an acute-care hospital affects social relationships. In this study, family members’ social interaction and support to each other was limited to their own family and no participant mentioned a need for support from other patients or their visiting family members. Family members had a need for social interaction on their own terms, and being able to visit the sick person at any time was crucial, with privacy described of great importance in allowing families to interact. These results are in line with other research, indicating that care environments can support social interaction between patients and family members and therefore also have the potential to increase mutual social support (Rigby et al., 2010 anonymized reference; Robinson et al., 2014; Ulrich et al., 2008), whereas inflexible visiting hours and multiple-bedded rooms add to family members’ discomfort. Our findings underline the importance of sharing everyday life and activities, e.g., meals, coffee and conversations, at the EoL care unit. Being able to sustain a personal rhythm at the EoL adds to everydayness as described by Rasmussen and Edvardsson (2007) in their study from different care contexts. However, our research suggests that everydayness seems equally important when the EoL care setting is an acute-care hospital, and family members can be seen as an important facet of everydayness.

Much is written about staffs’ attitudes and manner in relation to patient and family wellbeing; however it is notable how integrated this was in family members descriptions of their experiences of the care setting. Our results suggest that staff behaviours were strong signals to family members—whether the family members were seen as sources of knowledge, able to provide valuable support to the sick person and thereby also to staff, or as additional burdens adding to staff work load. Family members in this study described the importance of knowing what to expect and why, especially as curative and palliative care often coexisted and sometimes collided in the acute-care setting, and conversations about the upcoming EoL of the severely ill person did not always occur. Both Heyland et al. (2006) and Stajduhar et al. (2011) point to how lack of information can lead to family members’ guessing and struggling to understand the current situation.

Another finding related to family members’ need for guidance on how to act in and use the care unit. Feelings of uncertainty also seemed to increase when family members did not know who to approach about different questions, and asking for information was sometimes described as demanding courage and strength. In contrast, stories about encounters characterized by humour and laughter seemed to strengthen family members’ abilities to handle unfamiliar and sometimes frightening situations. A number of authors have emphasized that actively approaching and communicating with family members has a strong impact on their experiences of EoL care (Dose et al., 2015; Dosser & Kennedy, 2012; Heyland et al., 2006; Spichiger, 2009; Stajduhar et al., 2011), and our results suggest that staff contributed to whether environments were perceived as supportive or not.

### Methodological strengths and considerations

Research during ongoing care at the EoL is challenging due to short inpatient stays and the vulnerable situation of the participants. Difficulties in recruiting eligible family members for the study may be related to this lack of long-term contact and resulting unfamiliarity with patients and families, but perhaps also related to the ability to identify dying persons. Another issue was gatekeeping, as staff did not always allow family members to decide for themselves about participation in the study, perhaps as an effort to avoid overburdening both patients, families, as well as themselves. Approaching family members in vulnerable situations with research questions demands consideration and empathy, but their stories are important for developing healthcare that better suits their needs during difficult, life-changing situations (Gysels, Shipman, & Higginson, 2008). Motives given by family members for participating, included appreciating the method of data collection using participant-generated photographs, and the possibility to participate in research in an area they saw as important, in hope of contributing to change. To our knowledge, this is one of the first studies with family members in EoL care in acute-care units using photo-elicitation. However, photo-elicitation was also challenging for family members, as it demanded willingness to be subject to attention from others, as a few participants were approached and questioned by staff when they took photographs.

We found participant-produced photographs with subsequent interviews to be a useful approach for generating rich research data; we also noted that, as Pain points out (2012), it seemed to empower the participants and decrease the power imbalance between researcher and participant, as the latter set the agenda for the interview. Another strength in this study was that our data were generated during ongoing care and the participants’ stories did not rely on memories, which retrospectively might become more positive or negative. The participants actively searched and chose their motives with a camera in hand, in a more or less unfamiliar environment, later sharing their stories and thus giving us insight into their experiences, and highlighting the meaning of large and small things important to them. Various strategies were used to pay attention to quality throughout the research process; sampling to obtain breadth and variation through two information-rich data sources, validating our interpretive claims in frequent discussions of interviews, analysis and findings among authors, and making our interpretations visible by verbatim excerpts and photographs (Thorne, 2016). While this qualitative study was based on a limited sample of nine participants in two hospitals in the same country, we argue that nevertheless, the results may be relevant for a broader range of care contexts.

## Conclusion and implications

This study provides insights into family members’ experiences of acute-care environments during ongoing EoL care. It is already acknowledged in the literature that providing an aesthetic and comfortable environment has an impact on several outcomes relevant for patients’ wellbeing (e.g., Ulrich et al., 2008) and our study suggests that this may also be the case for family members. Aesthetic, multi-sensorial impressions, space for privacy that facilitates social relationships, and the way staff and the environment meet and guide family members, have the potential to add to experiences of both strain and wellbeing. These aspects can contribute to whether the environments are perceived as supportive or not.

Virdun et al. (2017) argue that many family members’ needs are still not met in acute-care settings, and how to achieve optimal EoL care remains a challenge. One relevant future research question is; how can expertise, ideas and insights from patients, family and staff be used in participatory processes to achieve more supportive environments in acute care? One of the ways these data have already proven relevant for clinical practice is through their use as discussion triggers in a process inspired by Experience-Based Co-Design (Bate & Robert, 2006) to achieve change in an EoL care setting, based on experiences, expertise and joint goals of patients, family members and staff. We combined data from all three perspectives to develop short films, and have found that the visual data complemented with interview text, has been a particularly powerful means for generating reflections and discussions. These films are made available to the general public including staff at the research program’s homepage (anonymized reference). Both methods and results of this study can thus be used to discuss and work with ideas to support change processes and promote the development of more supportive care environments for family members in acute-care settings during EoL care.
